# 4D flow MRI enhances prototype testing of a total artificial heart

**DOI:** 10.1038/s41598-025-18422-y

**Published:** 2025-09-15

**Authors:** Twan Bakker, Azad Najar, Thomas Finocchiaro, Ina Laura Perkins, Jonas Lantz, Tino Ebbers

**Affiliations:** 1 Department of Health, Medicine and Caring Sciences, Linköping University, Linköping, Sweden; 2 Center for Medical Image Science and Visualization (CMIV), Linköping University, Linköping, Sweden; 3 Scandinavian Real Heart AB, Västerås, Sweden; 4 Science for Life Laboratory, Department of Health, Medicine and Caring Sciences, Linköping University, Linköping, Sweden

**Keywords:** Magnetic resonance imaging, Biomedical engineering, Fluid dynamics

## Abstract

The shortage of donor hearts urges the need for total artificial hearts (TAHs). Existing TAHs cause complications like infection, stroke, and hemolysis due to adverse flow dynamics, but assessment of flow in a TAH is challenging. We investigated the feasibility of using 4D flow magnetic resonance imaging (MRI) to assess blood flow dynamics in the left side of a pulsatile TAH under representative physiological conditions. A prototype of a pulsatile TAH was modified using powder bed fusion and fused filament fabrication 3D printing techniques to function in an MRI environment. The TAH connected to an MRI compatible mock circulatory loop allowed variable physiological conditions (i.e. heart rate 80, 105, 120 bpm) and was scanned with two different velocity encodings. Flow patterns and turbulent kinetic energy were measured with high accuracy in a short measurement time and analyzed. Stasis and viscous energy loss in the artificial heart were found to be similar to healthy native hearts. Elevated turbulent kinetic energy was found in several areas, but values were well below those found in patients with valvular disease. Using 4D flow MRI in combination with 3D printing can facilitate assessment of flow dynamics in TAHs and enable a rapid iterative design process.

**Supplementary Information:**

The online version contains supplementary material available at 10.1038/s41598-025-18422-y.

## Introduction

The number of patients undergoing a heart transplant has steadily increased globally over the years to 8988 with an exception for 2020 when in general less transplants were performed amid the Covid-19 pandemic^1^. Despite this increase of surgical procedures, a persistent waiting list for heart donors remains with 2868 and 3453 people listed in the EU^2^ and the US^3^ respectively. For patients with biventricular heart failure, an option for solving this shortage could be to replace the failing human heart with a total artificial heart (TAH). TAHs are currently mainly used to bridge patients to transplantation. In order to use TAH as destination therapy, improvements are necessary to reduce blood damage and infection^4^.

Several initiatives are currently ongoing to improve TAHs for destination therapy. TAHs can be classified in two main categories: continuous-flow pumps and pulsatile pumps. The advantage of continuous-flow pumps is the small form factor, fitting smaller patients. However, for the majority of patients it also comes with a downside of acquired von Willebrand syndrome, in which platelet adhesion is reduced due to a protein deficiency believed to be caused by high shear stress, resulting in higher risks of internal bleeding^5,6^. Pulsatile TAHs could address this problem and currently one device is FDA approved for treatment (SynCardia, SynCardia systems, Tuscon AZ, USA)^7^. This TAH causes, however, high infection rates and major thromboembolic and hemodynamic events^8–10^. Another TAH (Aeson Carmat) has received the CE mark, enabling use as bridge to transplant in the EU^11^ and FDA approval for clinical trials in the US^12^. Due to the integrated hydraulic drive system, the Carmat TAH has a relatively large form factor. This makes implantation of the device more challenging, in particular for smaller patients. Several other TAH concepts arecurrently in different development stages^4^. Realheart TAH is a mechanically driven TAH undergoing animal trials and aims to deliver physiological blood flow to address various limitations of existing TAHs.

To minimize blood damage, assessment and optimization of the flow patterns in a TAH is crucial in the development phase of TAHs. For this, computational fluid dynamics (CFD) has been used on continuous-flow pumps and to a lesser extent pulsatile TAHs^13,14^. However, CFD analysis of a TAH, especially in a pulsatile TAH, is very challenging, due to the complex interaction of the flow with the surroundings, complex non-linear motion of membranes and valves, and a disturbed flow regime. Therefore, experimental data of the flow field is crucial to guide and validate the simulations. Pressure measurements alone would not provide a complete understanding of the flow, and thus other methods, providing more information about the flow, are desired. Experimental methods, as particle image velocimetry (PIV) and laser doppler anemometry, come with challenges in terms of creating an in vitro test setup, replicating the flow in a transparent model, and being limited to in-plane velocity measurements. This is particularly challenging for a complex system such as a TAH. Ultrasound and echoPIV prove similar challenges for an in vitro setup, where the complex moving geometry surrounded by air makes it difficult to place the measurement tool.

In recent years, 4D flow magnetic resonance imaging (MRI) has become popular in a clinical setting^15–18^. With this technique, the velocity field and turbulence intensity can be measured in a three dimensional and time resolved space, where thus the hemodynamic flow dynamics can be assessed^16,19^. It enables the evaluation of complex flow patterns like recirculation and Dean vortices, a double counterrotating vortex in the plane perpendicular to the main flow direction, also found in the aortic arch^20^. Blood stasis, a known risk factor for thrombus formation, has been quantified from 4D flow MRI derived velocity in the left atrium to estimate stroke risk^21^. Additionally, left ventricular efficiency in both patients and healthy controls has been analyzed by calculating viscous energy loss from the velocity field^22^. Furthermore, turbulence has been identified as a contributor to hemolysis through elevated shear forces in the fluid^23^, where turbulence can be detected and quantified by measures of turbulent kinetic energy (TKE). 4D Flow MRI has also been used to assess the flow in in vitro flow phantoms, on stenotic pipe flows, models of aortas, and mechanical heart valves, amongst others^24–26^. The technique has also been applied on continuous flow ventricular assist devices^27^ and on a pulsatile ventricular assist device (VAD)^28,29^. It has, however, not been used to investigate the blood flow in a multi-chamber flow driving pulsatile TAHs.

Here, we investigate the feasibility of using 4D flow MRI to assess blood flow dynamics in the left side of a pulsatile TAH prototype, the Realheart TAH, under representative physiological conditions. The objective was to examine local 3D flow features in complex geometries within the device, visualize the observed patterns, and calculate clinically relevant flow metrics that have been linked to blood damage to present insights that can improve the prototype in an iterative design process.

## Results

High quality velocity and turbulent kinetic energy data were acquired in the artificial heart (AH) with an acquired spatial isotropic resolution of 2.0 mm and effective temporal resolution of 23.0–24.9 ms in 9:02 min under physiological flow conditions for three different AH settings. Extending the drive shaft and replacing metal parts using 3D printing allowed for assessment of the whole field of view, except close to the mechanical heart valves, which were not replaced to assure functioning of the AH.

### General flow description

**Fig. 1:**
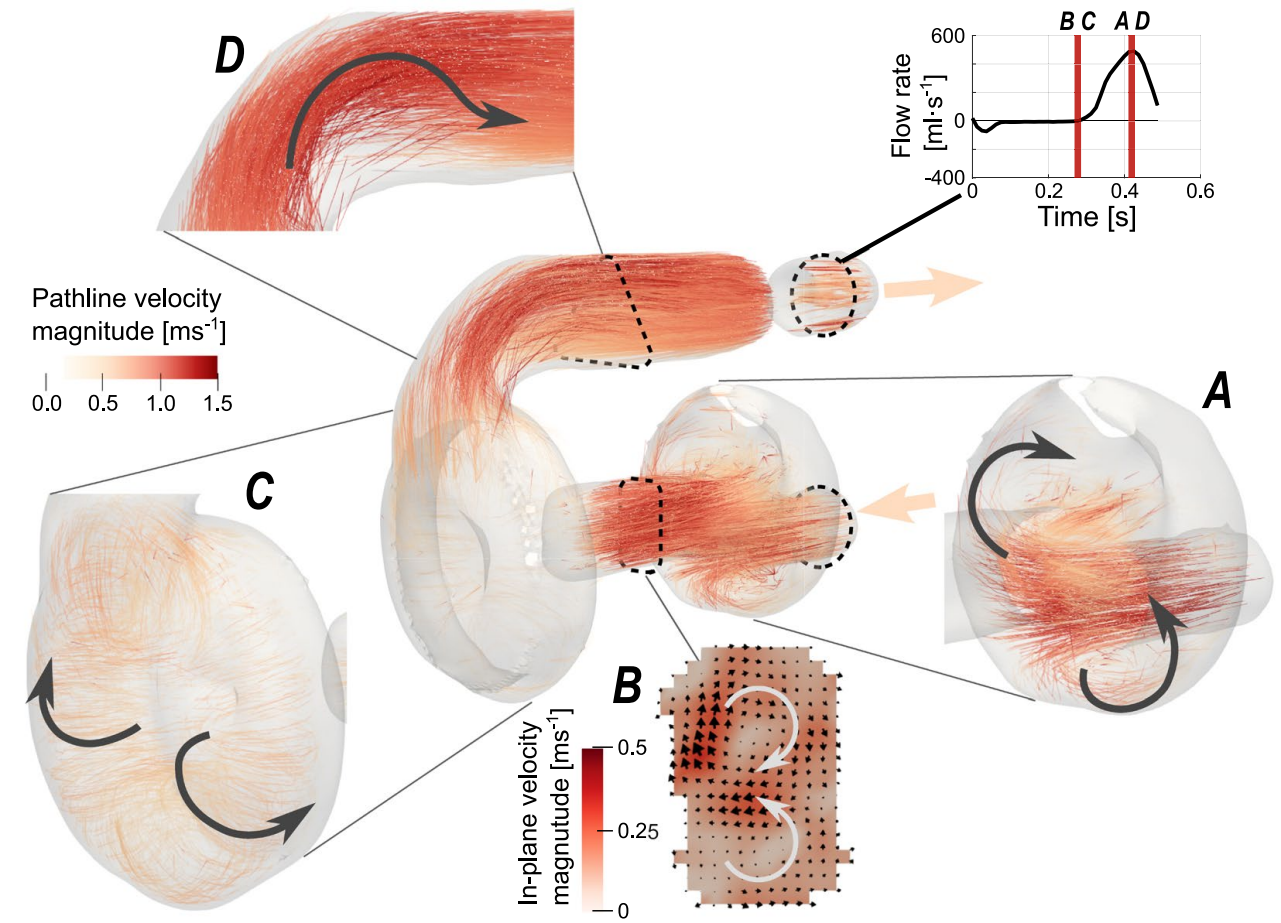
Overview of the flow analysis in the AH for 120 bpm. Overall representation of the full cardiac cycle in the center, with highlighted circulating flow patterns in various locations at specified time points in (**A**, **C**, and **D**). The Dean vortex through the AV-cylinder during end of diastole depicted in (**B**).

Pathline visualization of the acquired 4D flow MR dataset gave a comprehensive overview of the flow in the AH, and revealed complex flow pattern in the atrium, ventricle, and aortic bend. Flow enters the artificial heart from the venous compliance chamber into the atrium with a slightly eccentric profile, Fig. 1A, due to a 90-degree bend in the piping before the atrium. In the ventricular systolic phase, incoming flow enters the atrium striking the inner membrane and deflecting upwards and sideways of the conical atrial geometry. This created a recirculating flow in the atrium during the filling. As the atrium contracts in ventricular diastole, the flow is pushed through the atrioventricular AV-cylinder and mitral valve. Due to the high velocity magnitude in the initial phase of diastole, the flow appears to be unidirectional. This changes when the overall velocity in the AV-cylinder reduces, and here two strong counterrotating vortices appear in the plane perpendicular to the bulk flow direction, known as Dean vortices, Fig. 1B. Dean vortices have been observed in aortic flows in both patients as well as healthy volunteers^30^. Although the geometry of the AV-cylinder is straight, the flow moving from atrium to this cylinder comes in under an angle, causing a velocity gradient along the wall with the highest velocity in the center. This, in its turn, triggers a rotation perpendicular to the flow direction, resulting in these two vortices.

The mitral valve with a small angle between the leaflets deflects the fluid into an upward, downward, and center flow. As these components meet the opposing wall in the ventricle, its cup shape causes the formation of an all-around vortex ring and a somewhat doughnut pattern in the flow, Fig. 1C. The ventricle expands, and flow rotations slow down until the start of systole where the fluid is pushed to the outflow tract. Due to the 90-degree angle in this outflow tract, a recirculation occurs at the inner radius, effectively constricting the outflow, Fig. 1D. The fluid passes the aortic valve, which opened due to the pressure gradient build up in this phase, in a similar way as through the mitral valve. Once the valve is closed, the flow in the outflow tract settles until the new bulk of flow comes in the next cycle.

### Flow rate

**Fig. 2:**
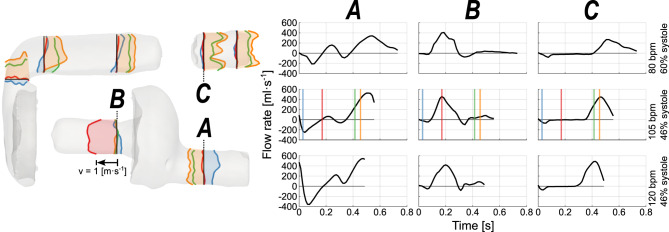
Axial velocity profiles through the centerline of several chosen locations and flow rates at the indicated locations for the three acquisitions. The velocity profiles shown on the left are from 105 bpm, colored by selected timeframes and scaled to the velocity magnitude, theblack line is the location of the measurement for each profile. The surface of the artificial heart is a static image from one of the depicted timeframes (blue) for reference. Flow rates in three distinct locations (**A**–**C**) show the difference between the three acquisitions from top to bottom. For 105 bpm the colored lines in the flow rate charts provide a time reference of the velocity profiles.

To ensure that the measurements were of sufficient accuracy, volumetric flow rates were measured at the inlet and outlet of the artificial heart, also indicated in Fig. 2. The measured difference in stroke volume between inlet and outlet were 4.6, 0.4, and 3.8 ml, with mean cardiac output was 4.1, 5.4, and 6.2 l/min for 80, 105, and 120 bpm respectively.

The flow rates over a cardiac cycle for different AH settings showed variation in both peak values and in profile (see Fig. 2). For the inlet, location A, the increase in heart rate resulted in both an increased peak flow rate and an increased backflow. In the AV-cylinder, location B, this shift in peak flow rate did not occur, as the maximum was similar for each acquisition, but instead the shape of the flow rate curve changed. It was observed that the difference in systolic ratio, 60% in the top row versus 46% in the middle and bottom row, created a more stretched inflow and outflow profile, whereas the diastolic phase, B top row, was as expected relatively shorter.

The Reynolds number at peak flow rate ($Re$) was calculated in the same locations as the flowrate. At the inlet, and outlet, the peak $Re$ was around 6700 and 7100 for 105 and 120 bpm respectively. For 80 bpm this value was lower at around 4200, which can be explained by the difference in systolic ratio for this heart rate acquisition. In the AV-cylinder, location B, the $Re$ for each acquisition was similar at around 7000, showing the impact a different systolic ratio has on the flow. It should be noted that this $Re$ is a very rough estimate as there were large flow rate differences over a cardiac cycle. This means that fully turbulent conditions would likely not have time to develop over the limited pipe-like sections in the artificial heart.

### Velocity profiles

The flow through the aortic valve was hypothesized to be the region where expected blood damage would occur, given high wall shear stresses on the valves as described in Bornoff et al.^13^. Since no measurement could be performed in the valves as it contains some metal causing a signal void in that region, the flow field before and after the valve was evaluated. From the velocity profiles through the outflow tract, Fig. 2, a clear difference was observed before and after the aortic valve in systole. Further upstream of the valve, a parabolic velocity profile with an asymmetry to the top was seen, slowly transitioning into a more uniformly distributed velocity profile before the aortic valve. Downstream of the aortic valve an expected change in the flow pattern was observed, with initially two major flow streams. Later in systole three clear peaks appeared due to the three openings caused by the leaflets, a pattern also observed for a different type of bi-leaflet valve in Kvitting et al.^25^ for steady flow. As the flow moves further through the outflow tract, the profile tends to continue to develop in a more similar profile as observed before the valve. Due to the chosen field of view in our measurement, we were unable to evaluate the velocity profile further downstream. Noticeable is the lower velocity in the center of the outlet conduit, implying that the fluid is not equally split between the three valve openings. Furthermore, the backflow in the inlet during systole at location A, Fig. 2, can be seen in blue. In addition, the effect of the smaller cross-sectional area of the AV-cylinder, which can be observed in red, led to higher velocities in that region compared to other regions.

### Quantification of stasis

**Fig. 3:**
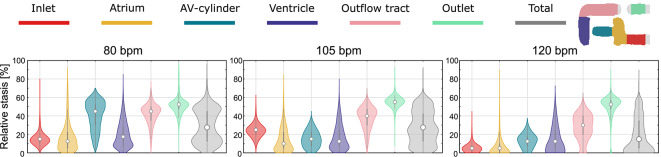
Violin plots showing relative stasis in various regions of the artificial heart and the entire AH for different heart rates. The colors represent the different regions where the larger grey violin plot indicates relative stasis in the entire AH. Median and quartiles are projected with a dot and grey lines respectively within the violin plots.

Slow moving flow, or stasis, can increase the risk of thrombus formation in that region ^31,32^. Stasis within a voxel was defined as a velocity magnitude below 0.1 m s^−1^ and relative stasis presented here is the time over the cardiac cycle this condition is met^21^. The median level of stasis, computed from the velocity field, in the entire artificial heart was observed to be decreasing with increasing heart rate, indicated with dots in the center of the violin plots in Fig. 3. Similarly, decreasing relative stasis with increased heart rate was observed in the atrium, ventricle, and outflow tract, which are also the largest evaluated volumes. Besides the median, the distribution of the data, emphasized with the violin plots, is observed to be more concentrated to the lower level of relative stasis for higher heart rate. No voxels were found with 100% stasis.

### Kinetic energy, viscous energy loss, and turbulent kinetic energy

In order to further assess blood flow dynamics, we studied the kinetic energy (KE), assessing locations and moments in the cardiac cycle with high velocities, viscous energy loss, assessing additional work required by the TAH, and turbulent kinetic energy (TKE), a measure of the intensity of the turbulent fluctuations in the velocities, which has been linked to blood hemolysis in numerous studies^23,33,34^.

**Fig. 4:**
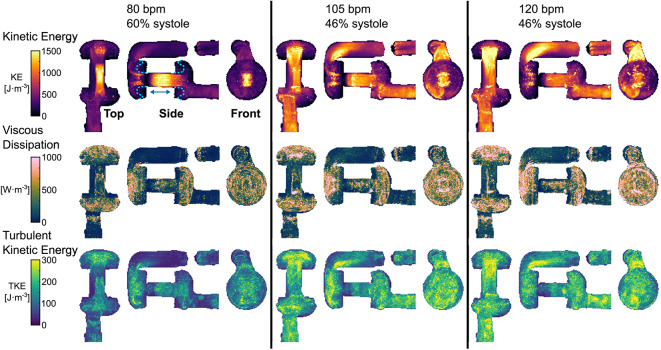
Maximum intensity projection (MIP) over the cardiac cycle of kinetic energy (KE) on the top row, viscous dissipation from which viscous energy loss is computed in the middle row, and turbulent kinetic energy (TKE) on the bottom row for the top, side, and front view of the evaluated AH. The blue dashed line in the 80 bpm side projection of KE represents the geometrical changes of the chambers between diastole and systole, with movement direction indicated by the arrow. The stroke volume was equal at 55 mL, with differences in heart rate of 80, 105, and 120 bpm, and systolic ratio of 60, 46, and 46% from the left to right column respectively.

In a maximum intensity projection (MIP), the maximum value over a cardiac cycle for all voxels in the direction of a slice was calculated. No filtering has been done on the MIPs that are presented in this study. Since KE is a function of velocity, the effect of higher velocities due to increased heart rate and same stroke volume were observed, Fig. 4 top row. The shorter diastolic time for the 80 bpm acquisition resulted in higher maximum KE in the AV-cylinder, whereas in the rest of the AH the maximum KE remained low compared to the other acquisitions.

The viscous dissipation, from which viscous energy loss is computed, shows MIPs that are dominated by noise in mainly the atrium and ventricle of the AH. The squared velocity gradients used in the calculation of viscous dissipation amplify the noise in regions where velocity is both high and not unidirectional. The regions with high values for viscous dissipation still show an increase in intensity for increasing heart rate.

For TKE, bottom row, increased values were mainly observed in the atrium, ventricle, and in the outflow tract after the 90-degree bend. The increased TKE in the ventricle and atrium could be explained by recirculations in these larger volumes where therefore larger velocity gradients occurred, see also the circulations in Fig. 1. The valuesalso showed that the sensitivity for maximum intravoxel velocity variation (IVVV) was close to the theoretical optimum, which was achieved with the selected velocity encoding (VENC) in this study. The effect of higher heart rates resulted in higher maximum TKE values throughout the artificial heart. The region after the mitral valve and aortic valve were initially hypothesized to show the maximum as the valves act, from a fluid dynamics point of view, as a constriction. It was therefore notable that the bend in the outflow tract showed an even higher measured TKE.

**Fig. 5:**
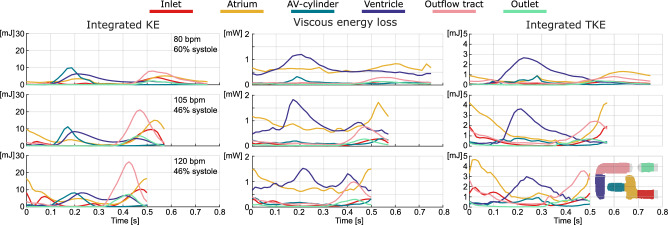
Measures of KE, viscous energy loss, and TKE in various regions of the AH over the cardiac cycle. KE integrated over volumes in the left column, viscous energy loss integrated over volumes in the center column, and TKE integrated over volumes in the right column. The stroke volume was equal at 55 mL, with differences in heart rate of 80, 105, and 120 bpm, and systolic ratio of 60, 46, and 46% from the top to bottom rows respectively. Note the different y-axis for each of the metrics, and different unit for KE, TKE, and viscous energy loss.

The metrics KE, viscous energy loss, and TKE integrated over the respective volumes provided insights on the effect of the different pump settings on the flow. In the KE measurements, Fig. 5 left column, there was an overall increase with increasing heart rate. The equal stroke volume and higher heart rate resulted in an increase in velocity from which KE is derived.

Viscous energy loss ($E_{L}{\prime}$), Fig. 5 middle column, shows that a higher heart rate results in an increase in losses for the different evaluated volumes. Similarly, the mean and maximum viscous energy loss over the entire volume is increasing with heart rate ($E_{{L_{mean} }}{\prime}$= 1.6, 2.3, 2.6 mW and $E_{{L_{max} }}{\prime}$= 2.3, 3.6, 3.5 mW). The atrium and ventricle are the largest contributors to the viscous energy loss in the entire AH. The outflow tract shows only a peak during systolic phase, during diastole the velocities are low resulting in relatively low viscous energy loss. As viscous energy loss is a volumetric integration of viscous dissipation, the distributed noise partially cancels out and can provide comparative value.

For TKE, Fig. 5 right column, the higher heart rate resulted in higher measured TKE in mainly the inlet, atrium, and outflow tract. Contrary, TKE in the ventricle was less affected by the heart rate alone, as seen for the low heart rate acquisition with different systolic ratio. This suggested that the combination of pump settings was more impactful in this specific region. As with KE, the effect of heart rate was most notable in the inlet, atrium, and outflow tract. The delay between the TKE and KE peak could be explained by the flow decelerating, causing the turbulent fluid structures to break into smaller vortices. Moreover, the maximum KE was around an order of magnitude higher than for TKE in each of the measured volumes. Additionally, the low TKE values in this graph were more prone to noise as described in Dyverfeldt et al.^24^.

### Shear stress distribution

**Fig. 6:**
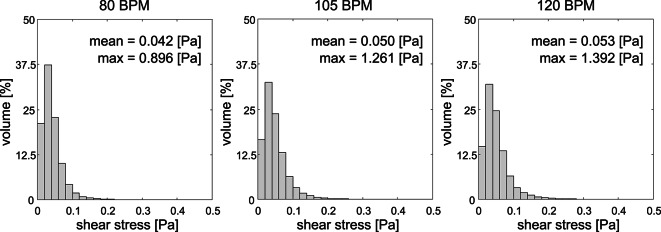
Histograms of the voxel-wise scalar shear stress distribution in the artificial heart for different heart rates. Each bin has a width of 0.02 Pa and the y-axis represents the percentage of the voxels in the AH between that scalar shear stress value. The plot represents the entire cardiac cycle. Mean and maximum shear stress values for each of the heart rates are reported in the plot.

High shear stresses can affect endothelial and the blood cells. Therefore, the distribution of the scalar shear stress in each voxel, computed from the velocity gradients, in the artificial heart was evaluated, Fig. 6. The distribution showed to be similar in shape for each heart rate with a clear skew towards the low shear stress values. The reported maximum and mean values indicate an increase of the scalar shear stress magnitude as heart rate increases. This behavior is similar to the observed values for viscous energy loss which is also derived from the velocity gradients.

### Outflow tract flow recirculation

**Fig. 7:**
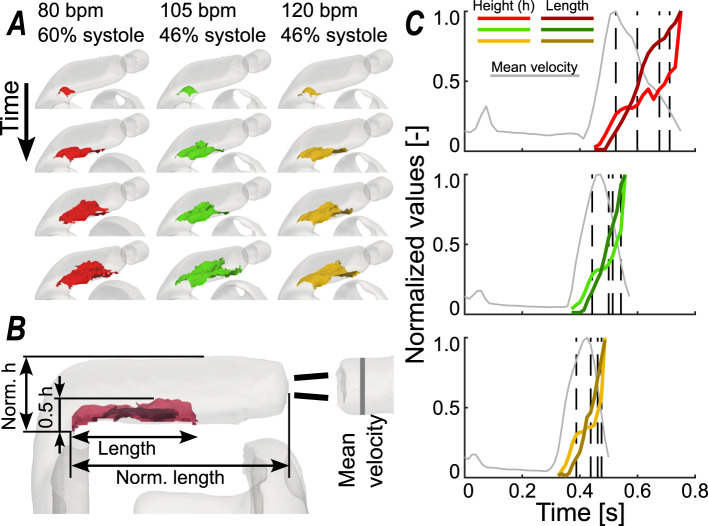
(**A**) Isosurfaces of the recirculation observed in the bend of the outflow tract, where the vortex propagates at the start of the systolic phase towards the aortic valve in length and height until the flow stagnates due to the valve closing. (**B**) the dimensions of the recirculation zone size, based on an isosurface where the velocity in axial direction (i.e. in flow direction) is zero. Included are the dimensions to normalize, and location where mean velocity is calculated. (**C**) the plot of the calculated recirculation height (brighter color) and length (darker color), for 80, 105, and 120 bpm top to bottom. The black dashed lines in C correspond to the timeframes of the illustrations in A.

The observed higher values of TKE in the outflow tract could be explained by the occurring flow recirculation, or separation, Fig. 7. The recirculation caused higher velocity gradients within the fluid in that location, and this consequently led to increased TKE values. At the beginning of systolic phase, the recirculation was seen to grow over the full width of the outflow tract increasing steadily in both length and height. This behavior was concluded in a fully recirculating flow at the end of the systolic phase when the velocity drops just before closure of the valve. To further assess this motion, the size of this recirculation zone, the height and length, were calculated. Given the pulsatile nature of the flow, the recirculation only spans the last part of the systolic period.

As the velocity in the outflow tract increases, the recirculation zone grew in a near linear fashion in terms of height and length, Fig. 7. It was observed that near the end of the systolic phase almost the entire outflow tract consisted of recirculating flow, as the determined recirculation height reaches the total height of the outflow tract. This happened before the valve was completely closed, as there was still net positive volumetric flow measured after the valve.

## Discussion

In this study, we show that 4D flow MRI, in combination with 3D printing, can measure relevant high-quality quantitative information about the intricate flow dynamics in a pulsatile AH operating under clinically representative operating conditions. More specifically, using this approach, both recirculation regions and turbulent flow patterns were discovered and could be measured, improving the understanding of the flow dynamics in the mechanical heart, and providing invaluable information for the iterative design process.

The usage of MRI to assess flow dynamics in the left side of the TAH required some adaptations to the TAH. Ferromagnetic metals can experience a force in the scanner, while all metals can result in image degradation. Most metal parts were replaced by 3D printed parts and the motor was placed outside the MRI system by extending the drive shaft. These changes were relatively easy to make and did not affect the functioning of the AH, with the components in contact with the fluid left unchanged. Adapting the AH to alternative techniques to assess the flow dynamic in the whole AH, as particle image velocimetry (PIV) and laser doppler anemometry (LDA), would have been considerably more challenging. In both Jung et al.^35^ and Lorenz et al.^36^, the experimental setup shows similarities to this study. Two fluid collection chambers wereconnected to the inlet and outlet of the device, and the pressure difference over the device was adjusted with a resistance. The ECG trigger to the MR was provided by the software controlling the pump and pressure data was collected away from the magnetic field.

The 4D flow MRI experiment resulted in high quality time-resolved 3D velocity and 3D TKE fields in relation to the short measurement time of only 9 min for the whole artificial heart. Flow rates were measured at different locations in the AH and showed a relatively small variance between inlet and outlet, supporting accuracy of the measurement method. As the background offset error in this study is minimal, indicated by zero flow when the valve is closed, due to the correction using a measurement with zero velocities, this variance seems to be dominated by limitations in spatial and temporal resolution. Both can be easily increased at the cost of a longer measurement time for experiments where higher accuracy is required. The outflow profile found in this study is comparable to the reported results for the same AH under a similar heart rate in a hybrid cardiovascular simulator^37^. The MR data provides less fluctuations at the end of the flow pulse but has a similar peak value. Moreover, the chosen VENC showed to result in an optimal TKE sensitivity for the regions with the highest measured values^24^, which are the most interesting TKE range.

The observed velocity patterns in the artificial heart agree with the reported velocity field of a computational fluid dynamics study on a the same AH prototype in Bornoff et al. and Kelly et al.^13,14^, with a comparable flow field in the atrium, AV-cylinder, ventricle and outflow tract. We do observe higher velocities in the AV-cylinder in our measurements than reported in the CFD study. This could be attributed to a slightly different heart rate and length of diastole. The measured inlet profile showed an asymmetry that can be explained by a perpendicular bend in the tubing of the test rig before the measured domain, while both prior CFD studies^13,14^ on the TAH defined a different, less sharp, inlet bend. However, the atrium seems to work as a flow regulator, effectively decreasing flow asymmetry, as the flow through the AV-cylinder towards the ventricle showed a uniform profile. In vivo we should expect a large variety of inlet conditions between patients, as the pulmonary veins will be connected differently given the variability in geometry, especially as most of the native atrium will be removed, but these findings suggest that this should not impact the flow after the atrium. The Realheart TAH allows constant filling of the atrium as there is no valve closing this chamber, this is contrasting to single chamber ventricular assist devices on which flow studies have been done^28,35,36^. The open filling resembles more physiological similarity to the native heart and possibly reduces the effects of pressure upstream of the device. Further downstream in the AH, the bend in the outflow tract after the ventricle creates a recirculation along the length of the outflow tract which continues until the aortic valve. These flow features have to some extent been reported earlier in CFD studies on this TAH^13,14^. However, this study provides more detail on these patterns. We do observe a higher maximum velocity in the evaluated artificial heart (2.7 m s^−1^) if we would compare it to the healthy physiological heart (0.94 m s^−1^)^38^, and other ventricular assist devices that have been evaluated with 4D flow MRI (~ 0.9 m s^−1^)^28,29,36^. This can be explained by the AV-cylinder and outflow tract of the artificial heart, which bear higher resemblance to the dimensions and geometry of the ascending aorta.

We observed flow vortices in both chambers of the artificial heart. In both cases, this is caused by impingement of the flow with the wall, forcing a circulation. Similar vortices have been found in the ventricle of the human heart^39,40^. This flow rotation is believed to enhance the mixing of the blood and to conserve momentum, resulting in a more efficient function of the heart^41^. Vortical flow is also believed to reduce blood stasis, which could decrease the risk for thrombus formation. The noise in the maximum intensity projection of viscous dissipation can be explained by an amplified effect of the squared velocity derivatives in combination with the low resolution from MRI. This makes viscous dissipation on itself a less useful metric to analyze flow, even though it should be noted that the resolution in this study is higher than normally used in clinical MR acquisitions. Viscous energy loss, computed as the volume integral of viscous dissipation partially handles the noise, as the effect of individual voxels diminishes. Viscous energy loss can be used comparatively with caution and was found to be comparable to 4D flow MRI studies in vivo. In the left ventricle of the artificial heart the maximum viscous energy loss ${E}_{{L}_{max}}{\prime}$ 1.2–1.8 mW compares better to healthy controls (around 1.1 mW) in contrast to patients with atrioventricular sepal disorder correction (around 3.8 mW)^42^. Similar results are found in the outflow tract where the ${E}_{{L}_{max}}{\prime}$ in the AH ranges 0.3–1.0 mW, which is slightly lower than healthy controls 1.5 mW and patients with hypertrophic cardiomyopathy 3.8 mW^43^.

Relative stasis showed a mean in the atrium (7–17%) and entire AH volume (21–30%) for the different evaluated heart rates that are comparable to atria in healthy young (25%) and older healthy controls (29%)^21^. Notice, however, that this Eulerian approach is not applicable in regions with dynamic geometries, as static voxels do not necessarily represent the same location in the moving fluid. Additionally, voxels that were not present throughout the entire cardiac cycle, typically close to the moving wall, were not included in this calculation.

We found, in this experimental study, slightly elevated values of turbulent kinetic energy in some areas of the total artificial heart. Turbulence kinetic energy (TKE) as a quantifiable measure is a good indicator of turbulence, which has been linked to platelet activation due to high shear stress^44,45^. Higher turbulence intensity in the AH did not just seem to be caused by high velocity, but rather by flow disturbances due to geometrical sharp angles or edges, e.g. from the valves or the 90-degree bend. However, peak TKE values downstream of the aortic valve were around 112–173 J m^−3^ for 80 to 120 bpm respectively, which is in agreement with peak TKE values found in a previous in vitro study (115 J m^−3^) on the same type of valve^25^. Since the mechanical valves contained metal, the loss of signal in those regions created an information void that limited evaluation of these locations. However, turbulence normally occurs at the breakdown of a jet. The highest peak TKE in the artificial heart for this study (around 240 J m^−3^ at 120 bpm) was observed in the bend of the outflow tract. This is comparable with peak TKE found in the ascending aorta of older healthy subjects, 223.5 J m^−3^^46^. The artificial heart operating at lower heart rates shows lower peak TKE (113 and 212 J m^−3^, for 80 and 105 bpm respectively) than healthy young volunteers^46^. TKE integrated over the outflow tract shows similar results, where the maximum value found in the AH over the cardiac cycle was 0.8–3.5 mJ for the different heart rates. This is lower than reported in the ascending aorta of healthy controls (3.7–6.4 mJ)^46–48^, although it should be noted that the integrated volumes were not provided, making direct comparison difficult. In the atrium we see a similar pattern, with the mean integrated TKE between 0.6 and 1.7 mJ in the AH, and 1–2 mJ^49^ in the healthy human heart. Here the volumes are comparable in size.The ventricle shows slightly higher integrated peak TKE in the AH (2.6–3.7 mJ) compared to healthy subjects in 4D flow MRI and CFD studies (1.5–2.5 mJ)^38,50,51^. This is in stark contrast though to diseased patients with high pulmonary regurgitation (6.0 mJ)^50^ or hypertrophic cardiomyopathy (7.1–14.8 mJ)^52^. This agrees with in vitro blood damage findings in Perkins et al.^53^, where lower hemolysis was measured compared to other TAH devices.

Our TKE measurements differ slightly from values found in CFD studies. However, the effect of TKE can easily have been misjudged in CFD studies on a pulsatile TAH due to the simplified turbulence models used, where a large portion of the turbulence is modeled and not simulated. With the 4D flow MRI technique we can extract this valuable data, which is otherwise only available through computer intensive scale resolved simulations, requiring vast amounts of computational resources, as a larger part of the turbulent flow is resolved and not modeled.

The scalar shear stress distribution shows a low magnitude of the shear stress field. In one CFD study on the Realheart TAH^13^, the scalar is presented for regions exceeding 17.5 Pa. In this 4D flow MRI study, no such high values were observed, with a maximum value of 1.4 Pa at 120 bpm. It should be noted that most of the high scalar shear stress values from the CFD simulation were found to be in proximity to the valve, which could not be evaluated in this study due to the signal void. In a study on the Carmat artificial heart^54^, using CFD simulations, the scalar shear stress was used to evaluate the safety of the device regarding blood damage, showing shear stresses under 1 Pa for more than 90% of the blood volume and maximum values exceeding 17.5 Pa is a small fraction of the total volume. The artificial heart in our study shows lower mean and maximum values. As the voxel size of the 4D flow MRI acquisition is large compared to the resolution used in CFD simulations, the velocity gradients in general are underestimated due to spatial averaging. This is also observed for computations in wall shear stress^55^, and therefore absolute values in this regard should be interpreted with caution and makes it therefore difficult to compare.

This study has some limitations. The signal voids at the valves created a discontinuity in the flow field and thus MRI-based estimations of pressure differences^56^ and washout^57^ of the artificial heart could not be evaluated. In addition, a more detailed velocity and turbulence mapping in closer proximity to the valve was therefore not possible, even though this study uses a spatial resolution that is higher than more commonly used in clinical 4D flow MRI. Future investigations with non-metallic heart valves could provide this comparative information. A blood mimicking fluid was used, as it is difficult to control blood especially for experiments with a longer duration. However, the fluid properties of the used fluid were physiologically representative to blood for a realistic flow in the rig. The rig itself does not completely represent a human vascular loop even though inlet and outlet pressures were set to physiological mean values. The pressure measurements during the data acquisition were likely damped by small air bubbles in the long catheter tubes. This, however, did not affect the mean pressures that were used to set the resistance in the flow loop, which is also further described in the supplementary material. Bulk flow or flow curve measurements in the test loop can be beneficial as validation of the measurements. In addition, hybrid mock circulation loops^58,59^ could provide a more realistic flow response than our test-rig.

The use of 4D flow MRI in combination with 3D printing can be a powerful complement to CFD simulation in the design process of TAHs. As most TAH designs contain active elements like membranes and prosthetic heart valves, simulation-based studies to evaluate design alternatives are computer intensive and challenging, especially in the setting of turbulence. Experimental studies using 4D flow MRI, comparing a range of different operating conditions and, in combination with 3D printed prototypes, testing various design alternatives, can guide CFD simulations. Using novel advanced turbulence mapping techniques in MRI it might also be possible to directly assess turbulent viscous stresses and predict hemodynamic blood damage^60^. Enhancing our comprehension of how flow dynamics influence blood-related ailments such as hemolysis and thrombosis holds the key to refining the design and operational settings of TAHs. This not only positions TAHs as a viable substitute for heart transplants but also enables fine-tuning the design of various cardiovascular devices and empowers optimization of surgical techniques and pharmaceutical treatments for cardiac diseases.

## Conclusion

We show that 4D flow MRI in combination with 3D printing can be used to assess velocities and turbulence in a pulsatile total artificial heart under representative hemodynamical conditions. This approach revealed the complex and changing flow structures in the pump throughout a cardiac cycle, including recirculation regions. Turbulence intensity marked new locations of interest that were not reported in earlier studies on the artificial heart. The measured stasis, viscous energy loss, and turbulence values in this study were found comparable to equivalent parts of the native heart. In addition, the measured scalar shear stress shows low mean and maximum values. The use of 4D flow MRI in combination with 3D printing can be a powerful complement to CFD simulation and blood testing in the design evaluation process of total artificial hearts.

## Methods

For this study, a pulsatile TAH was modified to function in an MRI environment and connected to a closed flow system, allowing variable physiological conditions such as blood pressure and peripheral resistance. MR velocimetry measurements, allowing for assessment of the velocity and turbulent intensity, were performed in a clinical 3 T MRI system.

### Pulsatile total artificial heart

**Fig. 8:**
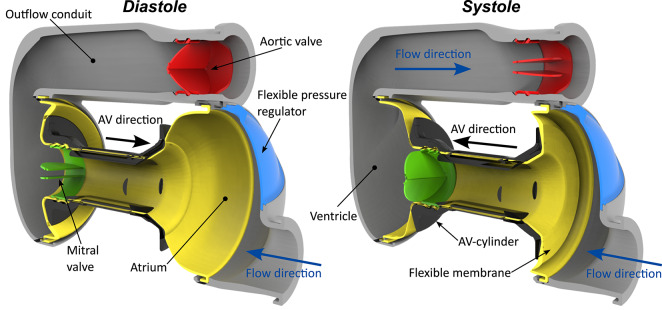
Illustration of the fluid domain of the Realheart AH indicating its different regions and important components in diastolic and systolic phase. The flexible membrane and pressure regulator are highlighted in yellow and blue. Diastole shows the expanded volume in the atrium and squeezed ventricular volume; the opposite is seen in systole. The mechanical mitral valve (green) and aortic valve (red) are shown in their respective opening and closing position during the indicated heart phase. The motor driven deformation of the membrane, by the stiff AV-cylinder (black), allows filling and emptying of the atrium and ventricle together with the closing and opening kinematics of the valves.

A prototype of the left side of a pulsatile TAH (v11c, legacy version; Scandinavian Real Heart AB, Västerås, Sweden) was used in this study. To replace the total physiological heart, two of these devices are combined. The artificial heart (AH) is a positive displacement pump modeled after the anatomy and function of the human heart with a fluid volume of 185 mL, shown in Fig. 8. It consists of components physiologically analogous to the biological heart with an atrium and ventricle, separated by a mechanical valve, functioning as atrioventricular valve on a moving plane cylinder (AV-cylinder). The AV-cylinder and chambers are connected to a flexible membrane, indicated in yellow, to allow movement and enables the chambers to change volume during the cardiac cycle. The ventricle and outlet to the aorta are separated by another mechanical valve of the same type (ON-X BMHV; Artivion, Kennesaw, GA, USA) to restrict backflow and to obtain a pulsatile outflow.

The pumping functionality is driven by moving the AV-cylinder, creating a systolic and diastolic phase during a cardiac cycle. The AV-cylinder is a stiff part that is connected to the motor of the pump and pushes against the flexible membrane. Moving the AV-cylinder towards the ventricle, in systolic phase, pulls in new fluid from the inlet, filling and expanding the atrium. Opposite motion, diastolic phase, causes a pressure gradient that opens the mitral valve and allows the fluid to move into the ventricle. During systole, the fluid is pushed towards the aorta due to the pressure build-up in the ventricle, as the mitral valve isclosed and aortic valve open.

**Fig. 9:**
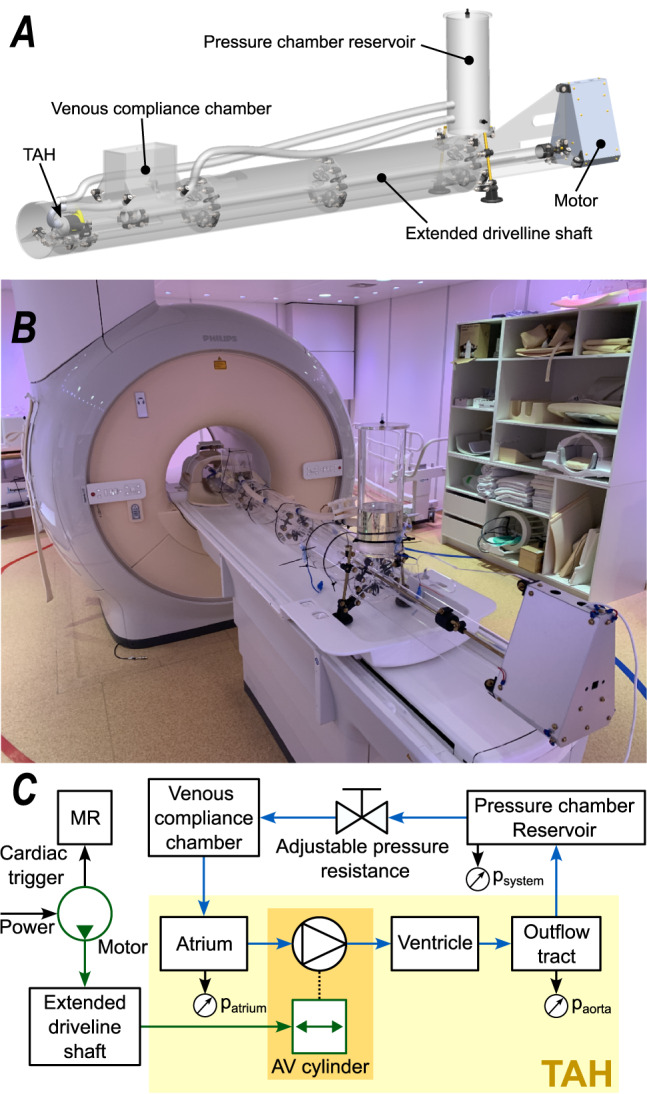
3D computer drawing representation of the experimental test rig (**A**), photograph of the experimental test rig within the MR room and scanner (**B**), and schematic (**C**) indicating the mechanical driveline green arrows, fluid loop blue arrows, pressure measurement locations, and communication to the MR system.

The Realheart artificial heart was adapted to the MR environment by removing ferromagnetic metals, which would be attracted by the magnet, and avoiding non-ferromagnetic metals, that would disturb the magnetic field resulting in signal voids in the vicinity of the metal. The fluid domain of the artificial heart or so-called blood unit, that is shown in Fig. 8, is supported by a housing created with powder bed fusion printing. The original TAH contains an electromotor in close vicinity of the AV-cylinder, which was relocated 2.5 m from the artificial heart, and thus the bore of the magnet, by extending the drive shaft, as shown in Fig. 9. A popular 3D printing method, fused filament fabrication (FFF), was used to create the AV-cylinder translating the motion of the motor to the flexible membrane. The non-ferromagnetic bi-leaflet mechanical heart valves were not replaced as this would affect the functioning of the artificial heart. These valves did cause an MR signal loss in their vicinity, but this was considered acceptable at this stage.

### Experimental flow loop

The TAH was integrated into a closed MR-compatible experimental flow loop wherein pressures representing the left side of the cardiovascular system could be regulated for a more representative flow field. The left side conditions were chosen as this side generally experiences larger hemodynamic forces acting upon the heart, and the release of a thrombus has more severe effects on an artificial heart recipient. The motor conveys its output through an axis consisting of brass and acrylic pipes, that are supported and guided in the test rig with printed guide wheels, where it connects to the artificial heart through a custom designed and powder bed fusion printed bracket. This connection to the atrioventricular cylinder results in a similar motion as in vivo and thus the function of the TAH continues to remain unchanged.

The flow circuit of the test rig was connected to the fluid domain of the TAH and comprises of silicon tubing, a pressure chamber functioning as a reservoir, and a venous compliance chamber equivalent of the atrial pressure, Fig. 9. The pressure within the system was regulated by the hydrostatic pressure in the reservoir and an adjustable resistance. Catheters were placed in the in- and outflow location of the artificial heart, at which clinically used pressure sensors were connected through pressure tubes, Fig. 9, and positioned at the same height as the measurement location. These pressures were used for post evaluation and quality control as well as for the live setup of the pressures within the system (see supplementary material for pressure curves).

The system was filled with a water glycerol mixture with a volumetric ratio of 1:0.53 to mimic blood, resulting in a density ($\rho$) of 1089 kg m^−3^ and dynamic viscosity ($\mu$) of 3.7 × 10^–3^ kg m^−1^ s^−1^. A contrast agent, 1 ml Gadobutrol (Gadovist) per 10 L fluid, was added to the fluid mixture to improve the velocity to noise ratio obtained from the MRI scanner.

Controlling the artificial heart was done with dedicated software developed for the clinical versions of the artificial heart (Scandinavian Real Heart AB, Västerås, Sweden). Functional parameters could be changed i.e., heart rate, stroke length, and systolic-diastolic ratio. Logs containing pressure measurements and feedback from the motor such as its reached position were exported.

For the experiments conducted in this study, the artificial heart was operated under various conditions summarized in Table 1. An average aortic pressure (${p}_{ao}$) of 110 mmHg, and average atrial pressure (${p}_{at}$) of 10 mmHg was established during the experiment to match the differential pressure used in the blood study in Perkins et al.^53^. The stroke length i.e., how far the AV-cylinder, Fig. 8, moves was set to 25 mm, resulting in a stroke volume of 55 mL.

**Table 1:** Settings of the artificial heart (AH) and test rig flow loop as used in this study.

Heart rate (BPM)	80	105	120
Systole/diastole (%)	60/40	46/54	46/54
Stroke length (mm)	25	25	25
Stroke volume (mL)	55	55	55
Average aortic pressure (mmHg)	110	110	110
Average atrial pressure (mmHg)	10	10	10

### 4D flow MRI acquisition and post-processing

The flow dynamics measurements in this study were obtained with a time-resolved three-dimensional MR sequence, popularly called 4D flow MRI, on a Philips Ingenia 3.0 Tesla system (Philips Medical Systems, Best, the Netherlands) using a 16-channel head coil. ECG-gating was used by an external trigger provided by the TAH software.

For this study an acquisition was conducted with a low VENC optimized for intravoxel velocity variation (IVVV) measurements, and high VENC for velocity measurements without phase wraps. Specific parameters used in the MRI acquisitions are shown in Table 2. Additionally, the measurement was repeated with the artificial heart turned off, to facilitate background correction offsets in the measurement regions^18^.

**Table 2:** Summary of the different MRI acquisition parameters.

Common parameters		
Number of encoding directions	3 (4-point asymmetric acquisition)	
k-space filling method	Cartesian	
Echo time (TE)	3.7 ms	3.2 ms
Repetition time (TR)	6.2 ms	5.7 ms
Flip angle (FA)	9°	
Velocity encoding limit (VENC)	70 cm/s isotropic	200 cm/s isotropic
Acquired spatial resolution	2.0 mm isotropic	
k-space segmentation factor	1	
Effective temporal resolution	24.9 ms	23.0 ms
Reconstructed spatial resolution	1.1 mm isotropic	
Reconstructed matrix size	140 × 140 × 100	
Reconstructed timeframes	40 (Δ14 ms per timeframe)	
Parallel imaging factor	SENSE factor 2.0 in AP and RL direction	
Time per acquisition (TA)	9:02 min	

The magnitude and phase images were reconstructed on the scanner and corrected for concomitant gradient fields and eddy currents. The background offset field was obtained by fitting a 2nd order 3D function to the fluid domain of stationary artificial heart acquisitions (i.e. AH turned off and no flow), extrapolated to the entire acquisition volume and then subtracted from the moving AH images. This allows correction of the entire domain, including regions that were not included in the stationary acquisition due to positioning of the AV-cylinder. Segmentation of the fluid domain was done semi-automatic by using a threshold on the MR magnitude data, whereafter manual adjustments were made in ITK-snap^61^.

### Flow analysis

Visualizations of particle traced pathlines, vortices, and 3D volume rendering were performed in ParaView (Kitware Inc., Clifton Park, NY, USA), and quantitative calculations on the MR data using MATLAB (The MathWorks Inc., Natick, MA, USA). Velocity data was evaluated using qualitative and quantitative metrics. Kinetic energy, a quantitative metric of the energy of the fluid as function of directional velocities (${u}_{i}$) and density ($\rho$), was computed as

1 $$KE = \frac{\rho }{2}\left( {u_{x}^{2} + u_{y}^{2} + u_{z}^{2} } \right)$$

Additionally, volumetric flow rates ($q$) were derived from the velocity component normal to the cross-sectional area ($u_{N}$) and the cross-sectional area ($A$)

2 $$q = u_{N} A$$

To estimate if the flow was laminar, transitional, or turbulent, the Reynolds number ($Re$) was calculated as

3 $$Re = \frac{{\rho q_{max} d}}{\mu A}$$

where $q_{max}$ is the maximum flow rate during the cardiac cycle through a cross-section with diameter, $d$.

Relative stasis ($r_{stasis}$) was calculated on a voxel level, as a percentage of time where the velocity magnitude within the voxel was below a threshold value of 0.1 m s^−1^ during a cardiac cycle^21^,

4 $$r_{stasis} = \frac{{n_{stasis} }}{{N_{tot} }} \times 100\%$$

where $n_{stasis}$ is the count of cardiac frames in which the voxel velocity magnitude was below the threshold, and $N_{tot}$ is the total amount of cardiac frames.

Viscous energy loss ($E_{L}{\prime}$) was calculated using the product of viscosity ($\mu$) and the sum of the voxel-wise dissipation ($\phi_{v}$)^22^,

5 $$E_{L}{\prime} = \mu \sum\nolimits_{i = 1}^{num\ voxels} {\phi_{v} V_{i} }$$

where $V_{i}$ is the voxel volume. Here the voxel-wise dissipation was calculated as

6 $$\phi_{v} = \frac{1}{2}\mathop \sum \limits_{i} \mathop \sum \limits_{j} \left[ {\left( {\frac{{\partial v_{i} }}{{dx_{j} }} + \frac{{\partial v_{j} }}{{dx_{i} }}} \right) - \frac{2}{3}\left( {\nabla \cdot {\text{v}}} \right)\delta_{ij} } \right]^{2} ,\ with\ \delta_{ij} \left\{ {\begin{array}{*{20}c} {0\ if\ i \ne j} \\ {1\ if\ i = j} \\ \end{array} } \right.$$

where $i$ and $j$ represent the three principal directions $x$, $y$, $z$, and ${\delta }_{ij}$ is the Kronecker delta. For the velocity gradients, a central difference scheme was used.

The MR magnitude data was used to compute the intravoxel velocity variation (IVVV), a measure of the fluctuating part of the velocity (Dyverfeldt et al.^24,62^). IVVV represents the squared normal components (${\sigma }_{ii}^{2}$) in three directions of the diagonal of the Reynolds shear stress tensor, from which turbulent kinetic energy ($TKE$) was derived as^63^,

7 $$TKE = \frac{\rho }{2}\left( {\sigma_{xx}^{2} + \sigma_{yy}^{2} + \sigma_{zz}^{2} } \right)$$

The scalar shear stress ($\tau$) was calculated using the product of viscosity and the norm of the strain rate tensor^64^,

8 $$\tau = 2\mu \left\Vert S_{ij} \right\Vert = \mu \sqrt {2S_{ij} S_{ij} }$$

where ${S}_{ij}$ is the strain rate tensor, similar as found in the voxel-wise dissipation of Eq. 6

9 $$S_{ij} = \frac{1}{2}\left( {\frac{{\partial v_{i} }}{{dx_{j} }} + \frac{{\partial v_{j} }}{{dx_{i} }}} \right)$$

In the outflow tract, the isosurfaces in Fig. 7 were calculated as location where the axial velocity is zero. The assumption was made that the isosurface goes through the core of the recirculation zone and that this is half of the total height of the recirculation zone. The length of the recirculation zone is defined as the length measured from the inner corner of the bend to the location near the wall where the axial velocity becomes positive again.

## Supplementary Information

Supplementary Information.

## Data Availability

The datasets generated and/or analyzed during the current study are not publicly available due to commercial sensitivity but are available from the corresponding author on reasonable request. Code availability: The code used for flow analysis in this study is openly available on Zenodo at DOI: 10.5281/zenodo.16940382^65^.
