# Supplementary material for: 4D flow MRI enhances prototype testing of a total artificial heart
Source: Sci Rep. 2025 Sep 15;15:32533. doi: 10.1038/s41598-025-18422-y (PMC12436633; doi:10.1038/s41598-025-18422-y)
Supplement: Supplementary file 1 — Supplementary Information. [file 41598_2025_18422_MOESM1_ESM.docx]

Supplementary material to:

**4D Flow MRI Enhances Prototype Testing of a Total Artificial Heart**

Twan Bakker^a,b^, Azad Najar^a,b,c^, Thomas Finocchiaro^c^, Ina Laura Perkins^c^, Jonas Lantz^a,b^, Tino Ebbers^a,b,d*^

^a^ Department of Health, Medicine and Caring Sciences, Linköping University, Linköping, Sweden
^b^ Center for Medical Image Science and Visualization (CMIV), Linköping University, Linköping, Sweden
^c^ Scandinavian Real Heart AB, Västerås, Sweden
^d^ Science for Life Laboratory, Department of Health, Medicine and Caring Sciences, Linköping University, Linköping, Sweden

# Supplementary material – Pressure measurements

During the MRI measurements pressures were recorded to match a physiological mean pressure difference over the TAH to the pressure used in Perkins et al.^53^

The outflow pressure measured for the different acquisitions, *Figure 10*, shows the response during the cardiac cycle. The wiggle within the pressure measurements for the different heart rates is likely caused by the rapid MR gradient changes and can be regarded as noise as the waveform remains measurable.

Unfortunately, the pressure recordings are likely damped, due to the long catheter tubes and, most importantly, air bubbles in the catheter tubes. The effect of the long catheter tubes has been described in a different study involving a test rig in the MR^36^ and is difficult to omit as the pressure sensors cannot be placed close to the magnetic bore. The effect of the air bubbles could have been minimized by better flushing of the catheter tubes. Additional experiments showed that the effect of the air bubbles did not affect the mean pressures, which were used to set the working conditions, but resulted in a smoothing effect on the minimum and maximum values of pressure. As the TAH is a positive displacement pump with two valves, the cardiac output is not expected to be affected significantly by a different pressure. This has also been addressed in a prior study by Fresiello et al.^37^. The time in which the valves close, and possible inertial effects contributing to cardiac output could be slightly affected by the pressure in the system.


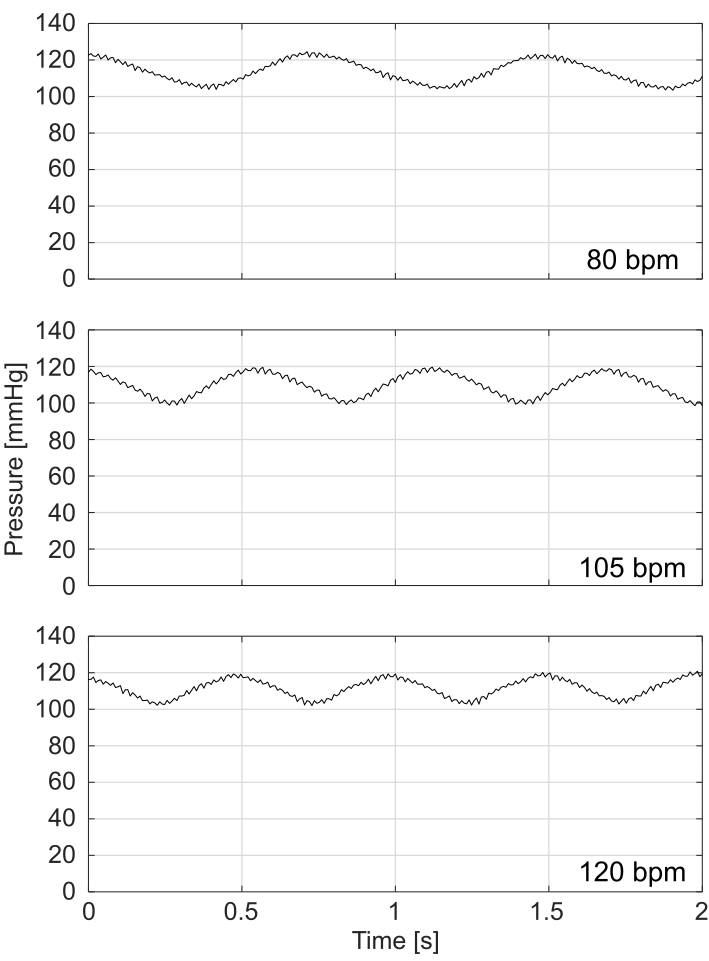


*Figure 10: Pressure readout at the outflow tract for the different acquisitions*
